# Attitude towards antiretroviral pre-exposure prophylaxis (PrEP) prescription among HIV specialists

**DOI:** 10.1186/1471-2334-13-217

**Published:** 2013-05-14

**Authors:** Vincenzo Puro, Antonio Palummieri, Gabriella De Carli, Pierluca Piselli, Giuseppe Ippolito

**Affiliations:** 1 UOC Infezioni emergenti e Centro di riferimento AIDS - Department of Epidemiology, National Institute for Infectious Diseases “Lazzaro Spallanzani” – IRCCS, via Portuense, 292, 00161, Rome, Italy; 2 Scientific Direction, National Institute for Infectious Diseases “Lazzaro Spallanzani” – IRCCS, Rome, Italy

**Keywords:** HIV pre-exposure prophylaxis, Antiretroviral drugs, HIV prevention, HIV healthcare providers, Health knowledge, Attitudes, Practice

## Abstract

**Background:**

To investigate perceptions and attitude to prescribe Pre-Exposure Prophylaxis (PrEP) among HIV specialists.

**Methods:**

A questionnaire developed through a Focus Group and literature review was administered to a convenience sample of HIV specialists during educational courses in two Regions and an online survey in February-May 2012. Participants were classified as having a positive or negative attitude according to their willingness to prescribe PrEP. Demographic and working information, experience with HIV-infected patients, information and provision of antiretrovirals to uninfected persons, self-reported knowledge, perceptions and concerns regarding PrEP were assessed. The association between a different attitude towards PrEP prescription and selected characteristics was assessed through univariate and multivariate regression analysis.

**Results:**

Of 311 specialists, 70% would prescribe PrEP, mainly to serodiscordant partners (64%) but also to people at ongoing, high risk of HIV infection (56%); 66% advocated public support of costs. A negative attitude towards PrEP was significantly associated with lack of provision of information on, and prescription of, antiretroviral post-exposure prophylaxis; specialists with a negative attitude believed behavioural interventions to be more effective than PrEP and were more concerned about toxicity. Overall, 90% of specialists disagreed regarding a lack of time for engaging in prevention counselling and PrEP monitoring; 79% would welcome formal guidelines, while those with a negative attitude did not consider this advisable.

**Conclusions:**

Although conflicting attitudes appear evident, most specialists seem to be willing, with guidance from normative bodies, to promote PrEP within multiple prevention strategies among vulnerable populations. More scientific evidence regarding effectiveness could overcome resistance.

## Background

The impressive improvements in the treatability of HIV infection have not been paralleled by similarly remarkable improvements in the effectiveness of HIV prevention strategies; therefore, more successful approaches are urgently needed.

In the last years, the emphasis in the field of research on prevention has shifted to the development of combination prevention packages in which structural, behavioural, and biomedical interventions are implemented concurrently [1].

Biomedical prevention strategies include using antiretrovirals (ARV) treatment as prevention (TasP), i.e. treating HIV-infected patients, regardless of clinical status, to curb transmission. However, gaps in the continuum from HIV diagnosis, through linkage to care, uptake and adherence to antiretroviral therapy, are substantially limiting its actual impact [2,3].

More recently, the use of ARV for pre-exposure prophylaxis (PrEP) among high-risk persons without HIV is emerging as one additional strategy to curtail the HIV epidemic.

While there have been significant studies suggesting the high potential efficacy of PrEP, although strongly related to adherence [4-7], others were halted early due to no difference between placebo and PrEP arms [8-10], again perhaps due to difficulties with adherence.

In any case, several questions arise about PrEP delivery in the real world. In particular, it seems necessary to understand the point of view of the providers on the actual implementation of PrEP in their clinical context, as well as to investigate how PrEP will be perceived by the communities of individuals at high risk.

In Italy, the care for HIV-infected patients, counseling, and testing were almost exclusively carried out by specialists in Infectious Diseases; the same is true for the provision of ARV. In addition, these HIV specialists have built up extensive expertise in prescribing ARV for post-exposure prophylaxis (PEP) to prevent occupational and sexual acquisition of HIV infection [11,12].

Although PrEP has not been regulated or tested at a national level so far, HIV specialists are likely to be the first prescribers of PrEP [13], and might have patients who require it. We therefore decided to investigate perceptions regarding PrEP through a survey of HIV specialists, to assess their attitude to prescribe and monitor PrEP use, and to identify factors predictive of a positive attitude towards PrEP prescription.

The survey was conducted between February and May 2012, when the results of CAPRISA and iPREX trials had been published [4,5], Partners and TDF2 studies had been presented [6,7], and the inconsistent results of FEM-PrEP and VOICE studies were already available [8,10].

## Methods

### Participants, focus group and survey instrument

To develop the questionnaire, a focus group [14] discussing PrEP potential impact on HIV prevention was conducted in January 2012 at the “Lazzaro Spallanzani” National Institute for Infectious Diseases in Rome. On the basis of the existent literature on PrEP, a Focus Group Interview Guide was developed focusing on the following main areas: knowledge on PrEP trials, ideal target populations, attitudes and concerns about this potential new biomedical HIV prevention tool.

A convenience sample of 10 internal HIV specialists voluntarily participated in the focus group. The focus group was guided by a physician (V.P.) and a psychologist (A.P.), lasted 2 h30 and was digitally audio-recorded, verbally-transcribed, analysed, and the main themes were extracted.

Then the Fenway Institute group was contacted [15], and part of their original survey tool, kindly provided, was incorporated in the first version of the questionnaire after adaptation.

In February 2012, a pilot study was conducted administering the questionnaire to HIV specialists before the beginning of an HIV educational course, dedicated to PEP and PrEP, in Tuscany.

After revision, which determined the addition of four items, the questionnaire was administered to other specialists in Tuscany, and in Latium before analogous courses; denominators in the analysis may vary accordingly.

In May the survey was put online through LimeSurvey®, and an e-mail invitation was sent to contacts included in a professional listserve of the Italian Society of Infectious and Tropical Diseases, soliciting the specialists to participate in the project through the Web link.

A copy of the questionnaire is attached to this article (Additional file 1).

The study was approved by the National Institute of Health (Istituto Superiore di Sanità), and is in compliance with the World Medical Association Declaration of Helsinki.

Participation was voluntary, no financial incentive was offered. Both the paper-and-pencil and online surveys were completely anonymous, did not include personal/sensitive data, and according to the Italian legislation, did not specifically require to be approved by an Ethics Committee.

### Measures and variable definitions

The specialists were asked if they would ever prescribe PrEP, and to whom, among a number of possible target populations. Participants were classified as having a positive attitude if they would consider prescribing PrEP at least in some cases, or a negative attitude if they would not prescribe PrEP in any case.

Demographic and working information included gender, age class, length of service, workplace (non teaching hospital or university hospital/research institute) and prevailing activity (ward/inpatients, outpatient clinic/day hospital).

Experience was investigated through the years of work with HIV patients, the total number of HIV patients cared for and the number of HIV tests prescribed in the last month.

PEP or PrEP prescription to HIV-uninfected persons, and pre-emptive provision of information on PEP to patients were investigated.

Participants were asked to self-evaluate their own knowledge regarding PrEP.

Perceptions regarding PrEP were assessed through participants’ agreement with a series of statements. Whether participants would ascribe the costs of PrEP to the National Health System (NHS) was also asked.

 Moreover, after the pilot study, an hypothetical scenario of “a serodiscordant couple where the HIV-infected partner is not eligible for starting ARV according to national guidelines”, was presented and participants were asked about their attitude towards counselling on safe sex only vs. the additional use of ARV to protect the uninfected partner.

Finally, participants were asked whether they would consider appropriate that formal guidelines were issued to guide PrEP prescription or a clinical trial started in which to enrol patients.

### Statistical analysis

The association between a positive attitude towards PrEP prescription and selected characteristics was assessed by odds ratio (OR) and their 95% confidence intervals (CI). A multivariable logistic regression analysis was conducted to examine the association between the set of significant bivariate variables and positive attitude to PrEP; the adjusted OR (MLR-OR) were assessed selecting, through backward elimination (p > 0.20 as drop-out criteria) for the final model, all those variables found to be significantly (p < 0.25) associated with the outcome at the univariate analysis forcing age and gender; the Hosmer-Lemeshow goodness of fit test was used to assess model fit [16]. Data management and analysis were performed using IBM®-SPSS® Statistics version 19.0 (Chicago, IL).

## Results

### Focus group

Most focus group participants reported a “not detailed” knowledge about the results of published PrEP trials, and expressed concerns regarding PrEP use. The main concerns that emerged were: duration of use, side effects and long term toxicities; ARV resistance; a possible increase in sexually transmitted infections (STI); and the problem of monitoring adherence.

The high costs of drugs to be used for PrEP in HIV negative individuals and the possible competition with the availability of funding for ARV treatment of infected persons and for other preventive measures such as information campaigns, were considered difficult-to-resolve ethical issues.

The specialists did not find an agreement on PrEP target populations. The majority agreed to consider PrEP in case of stable serodiscordant couples who wish to conceive, in particular when the HIV negative partner is the woman, while they would be more careful in offering PrEP to individuals who report high-risk sexual behaviours, mainly for the fear of sexual disinhibition.

Finally, participants expressed the need for institutional recommendations, and their willingness to participate in PrEP trials.

### Study population

The questionnaire was returned by 311 physicians (overall response rate 40%), 40% of whom completed the online survey.

Thirty per cent of specialists (n = 95) would not prescribe PrEP in any case. Of the remaining 216, 81% would prescribe it to high-risk subjects in some circumstances, and 93% to serodiscordant partners. Main target populations were men who have sex with men (MSM) not using condoms (55%), and serodiscordant individuals exposed to a viraemic partner (45%); PrEP to women for conception, regardless of the viral load of their partner, accounted for 71% (Table 1).

**Table 1:** Attitudes towards pre-exposure prophylaxis (PrEP) prescription among 311 HIV specialists

***Based on the available evidence, do you think PrEP should be offered, and if yes, in which of the following situations?***			
	**N (%)**		
No	95 (30.5)		
Yes	216 (69.5)		
*Participants who think that PrEP should be offered* (N = 216)			
**Offer PrEP to some groups of people at higher risk:**^**a**^			
Injection drug users	45 (20.8)		
	***Not using condoms***	***Always***	***Total***
	**N (%)**	**N (%)**	**N (%)**
Men who have sex with men (MSM)	118 (54.6)	34 (15.7)	152 (70.3)
Persons with sexually transmitted infections	90 (41.7)	35 (16.2)	125 (57.9)
Persons with multiple partners	91 (42.1)	40 (18.5)	131 (60.6)
Sex workers/Transactional sex	84 (38.8)	47 (21.8)	131 (60.6)
**Respondents**	174 (80.6)		
**Offer PrEP to HIV-uninfected partner in serodiscordant couples:**^**a**^			
	***Viremic partner***	***Always***	***Total***
	**N (%)**	**N (%)**	**N (%)**
Men in heterosexual couples…	86 (39.8)	62 (28.7)	148 (68.5)
Women in heterosexual couples…	98 (45.4)	73 (33.8)	171 (79.2)
Men in MSM couples…	97 (44.9)	73 (33.8)	170 (78.7)
Women for conception…	71 (32.8)	82 (38.0)	153 (70.8)
Men for conception…	72 (33.3)	60 (27.8)	132 (61.1)
**Respondents**	200 (92.6)		

^a^ Percentages may exceed 100% as participants checked all that applied.

Demographic and working characteristics of the study population, experience with HIV-infected patients, practice and knowledge, overall and according to the attitude towards PrEP prescription, are shown in Table 2.

**Table 2:** Factors associated with positive attitude towards PrEP prescription among HIV specialists

	**Attitude towards PrEP prescription**		**Total (N = 311)**	**Odds-ratio for positive attitude towards PrEP prescription**	
	**Positive (N = 216)**	**Negative (N = 95)**			
	**N (%)**	**N (%)**	**N (%)**	**OR (95% CI)**	**MLR-OR (95% CI)**
**Demographic characteristics and work history**					
Gender					
Male	122 (56.5)	52 (54.7)	174 (55.9)	1	1
Female	94 (43.5)	43 (45.3)	137 (44.1)	0.93 (0.57-1.51)	0.85 (0.50-1.45)
Age Class					
≤40	33 (15.3)	18 (18.9)	51 (16.4)	1	1
41-50	51 (23.6)	26 (27.4)	77 (24.8)	1.07 (0.51-2.25)	0.68 (0.30-1.56)
>50	132 (61.1)	51 (53.7)	183 (58.8)	1.41 (0.73-2.73)	1.07 (0.51-2.25)
Length of service (years)					
< 10	35 (16.2)	20 (21.1)	55 (17.7)	1	
10-19	71 (32.9)	35 (36.8)	106 (34.1)	1.16 (0.59-2.29)	
> = 20	110 (50.9)	40 (42.1)	150 (48.2)	1.57 (0.81-3.03)	
Workplace					
Non teaching hospital	129 (59.7)	58 (61.1)	187 (60.1)	1	
University/Research institute	87 (40.3)	37 (38.9)	124(39.9)	1.06 (0.65-1.73)	
Prevailing activity					
Ward/Inpatients	116 (53.7)	53 (55.8)	169 (54.3)	1	
Outpatient clinic/Day hospital	100 (46.3)	42 (44.2)	142 (45.7)	1.09 (0.67-1.77)	
Survey					
Online Survey	69 (31.9)	55 (57.9)	124 (39.9)	1	1
Regional HIV education	147 (68.1)	40 (42.1)	187 (60.1)	**2.93 (1.78-4.82)**	**3.25 (1.79-5.91)**
**Participants’ experience with HIV-infected patients**					
Onset of activity with HIV patients					
HAART era	57 (26.4)	34 (35.8)	91 (29.3)	1	
Pre-HAART era	159 (73.6)	61 (64.2)	220 (70.7)	1.55 (0.93-2.61)	
HIV-infected persons currently followed					
< =5	45 (20.8)	19 (20.0)	64 (20.6)	1	
6-50	61 (28.2)	28 (29.5)	89 (28.6)	0.92 (0.46-1.85)	
>50	110 (50.9)	48 (50.5)	158 (50.8)	0.97 (0.51-1.82)	
HIV tests prescribed in the last month					
< =5	109 (50.5)	46 (48.4)	155 (49.8)	1	
6-20	77 (35.6)	33 (34.7)	110 (35.4)	0.98 (0.58-1.68)	
>20	30 (13.9)	16 (16.8)	46 (14.8)	0.79 (0.39-1.59)	
**Practice**					
Antiretrovirals prescription to HIV negative individuals					
No	26 (12.0)	30 (31.6)	56 (18.0)	1	1
Yes	190 (88.0)	65 (68.4)	255 (82.0)	**3.37 (1.86-6.12)**	**2.43 (1.29-4.61)**
Pre-emptive information on Post-Exposure Prophylaxis					
No	65 (30.1)	44 (46.3)	109 (35,0)	1	1
Yes	151 (69.9)	51 (53.7)	202 (65,0)	**2.00 (1.22-3.30)**	**1.72 (1.01-2.92)**
**Knowledge**					
Self-evaluation of PrEP knowledge					
Poor	73 (33.8)	27 (28.4)	100 (32.1)	1	1
Sufficient	72 (33.3)	42 (44.2)	114 (36.7)	0.63 (0.35-1.14)	0.87 (0.46-1.66)
Good	71 (32.9)	26 (27.4)	97 (31.2)	1.01 (0.54-1.90)	1.75 (0.82-3.74)

*PrEP*, Pre-exposure prophylaxis; *HAART*, Highly Active Anti-Retroviral Treatment; *OR*, Odds ratio; *MLR-OR*, Multiple variable Logistic Regression – Odds Ratio; *CI*, Confidence Interval.

Boldface = p < 0.05.

The majority of the respondents were males, aged >50 years, working in non-teaching hospitals, with inpatients, for more than 20 years.

Most participants had experience of the use of ARV in HIV negative persons (255, 82%); 85% had prescribed less than 20 HIV tests in the last month.

Overall, self-evaluation of PrEP knowledge was reported as at least sufficient in 69% of cases. At univariate analysis, no differences were found between specialists with a positive or negative attitude towards PrEP prescription according to demographic characteristics, working experience, experience with HIV-infected patients, or self-reported knowledge.

A positive attitude was more frequent among those who participated in the HIV educational courses, had prescribed ARV to negative individuals to prevent HIV infection, and used to inform their patients previously on PEP. All these factors remained significantly associated with a positive attitude at multivariate analysis (Table 2). The model demonstrated adequate goodness of fit, Hosmer-Lemeshow χ^2^_8_ = 10.195, p = 0.252, and accounted for 17.2% of the variance.

Participants’ perception of PrEP and their main concerns, overall and according to attitude towards prescription, are shown in Table 3.

**Table 3:** Perception of PrEP among HIV specialists according to their attitude towards PrEP prescription

		**Positive**	**Negative**	**Total**	**OR (95% CI)**
		**216**	**95**	**311**	
I am concerned that PrEP will not be 100% effective (N = 295)	Agree	161 (78.9)	77 (84.6)	238	1
	Disagree	43 (21.1)	14 (15.4)	57	1.47 (0.76-2.85)
I am concerned about the potential side effects of PrEP (N = 297)	Agree	133 (64.9)	75 (81.5)	208	1
	Disagree	72 (35.1)	17 (18.5)	89	**2.39 (1.31-4.35)**
I feel uncomfortable prescribing drugs for new indications unless there is clear evidence of benefit (N = 293)	Agree	132 (65.0)	71 (78.9)	203	1
	Disagree	71 (35.0)	19 (21.1)	90	**2.01 (1.12-3.60)**
I am concerned about a low adherence to PrEP* (N = 159)	Agree	55 (59.8)	44 (65.7)	99	1
	Disagree	37 (40.2)	23 (34.3)	60	1.29 (0.67-2.48)
I do not have time to engage in prevention counselling and PrEP monitoring (N = 290)	Agree	22 (11.0)	7 (7.8)	29	1
	Disagree	178 (89.0)	83 (92.2)	261	0.68 (0.28-1.66)
The use of PrEP will cause patients to engage in riskier behaviours (N = 297)	Agree	145 (70.4)	73 (80.2)	218	1
	Disagree	61 (29.6)	18 (19.8)	79	1.71 (0.94-3.10)
The provision of PrEP will result in an increase in sexually transmitted disease incidence among patients (N = 296)	Agree	136 (66.7)	67 (72.8)	203	1
	Disagree	68 (33.3)	25 (27.2)	93	1.34 (0.78-2.31)
Encourage access to testing and care for HIV infection are more effective measures* (N = 160)	Agree	81 (87.1)	64 (95.5)	145	1
	Disagree	12 (12.9)	3 (4.5)	15	3.16 (0.86-11.68)
Non-biomedical HIV prevention interventions (behavioural) are more effective than PrEP (N = 290)	Agree	136 (67.7)	79 (88.8)	215	1
	Disagree	65 (32.3)	10 (11.2)	75	**3.78 (1.84-7.77)**
Non-biomedical HIV prevention interventions (behavioural) are safer than PrEP (N = 301)	Agree	183 (88.0)	84 (90.3)	267	1
	Disagree	25 (12.0)	9 (9.7)	34	1.28 (0.57-2.85)
The use of PrEP will result in less frequent HIV testing among patients (N = 299)	Agree	90 (43.7)	52 (55.9)	142	1
	Disagree	116 (56.3)	41 (44.1)	157	**1.63 (1.00-2.68)**
PrEP is too costly (N = 295)	Agree	159 (77.6)	78 (86.7)	237	1
	Disagree	46 (22.4)	12 (13.3)	58	1.88 (0.94-3.75)
The use of antiretrovirals for prevention will select for, and disseminate, antiretroviral drug resistance (N = 294)	Agree	135 (66.5)	68 (74.7)	203	1
	Disagree	68 (33.5)	23 (25.3)	91	1.49 (0.85-2.60)

Denominators are reported for each item as non respondents were excluded at univariate analysis; percentages have been calculated on respondents.

* reference sample with different denominator (N = 172); *PrEP*, Pre-exposure prophylaxis; *OR*, Odds Ratio.

Boldface = p < 0.05.

Regardless of their attitude, the large majority of respondents agreed that encouraging access to HIV testing and care, and behavioural interventions, are more effective (91% and 74%, respectively) and safer (89%), than PrEP; regarding PrEP possible effect on testing frequency, respondents split evenly.

Main concerns were, in order: efficacy, costs, increase in risk behaviours and STI, side effects, risk of drug resistance and adherence. In addition, 70% of HIV specialists felt uncomfortable in prescribing ARV for new indications in absence of clear evidence of effectiveness and safety, while 90% disagreed to lack time for engaging in prevention counselling and PrEP monitoring.

Regarding prescription barriers, specialists who would prescribe PrEP are less concerned by potential toxicity and use of drugs for new indications (OR 2.39 and 2.01, respectively); moreover, they are more likely to disagree that behavioural interventions could be more effective than PrEP (OR 3.78), or that PrEP could lead to a decreased attitude to test regularly (OR 1.63).

Overall, most respondents believed that NHS should sustain PrEP costs: entirely, in all (28%) or selected (9%) cases (i.e. conception), or partially, based on patient’s income (29%). Of the remaining, 31% would charge the patient, and 3% did not answer. Specialists who would prescribe PrEP are more likely to support NHS participation in covering PrEP costs (OR 4.63; 95% CI 2.74-7.84). Of the two items not included in the pilot session (denominator = 279 specialists), in the hypothetical scenario, of 271 respondents 55% would choose only counselling on safe sex to protect the uninfected partner, while 27% would add ARV to the infected partner, 8% PrEP to the negative partner, and 10% both.

Those with a positive attitude towards PrEP prescription are definitely more likely to add PrEP, alone or in combination with TasP, to prevent transmission (OR 4.46; 95% CI 1.78-11.16). As for the framework for PrEP prescription, 79% considered appropriate the issue of formal guidelines (not recommended 11%; 10% did not answer), and 60% the start of a multicenter trial (not recommended 22%; 18% did not answer). Those who would prescribe PrEP are more prone to provide it within the framework of a multicenter trial (OR 3.95; 95% CI 2.14-7.30; p < 0.001) and national/international PrEP guidelines (OR 3.37; 95% CI 1.51-7.51; p = 0.003).

## Discussion

In our survey, despite the multiple challenges identified, there was a prevalent positive attitude of HIV specialists towards PrEP prescription. Indeed, 70% of respondents would offer PrEP, mainly to serodiscordant partners (64%), especially women and MSM engaged with viraemic partners, but also (56%) to people at ongoing, high risk of HIV infection, primarily MSM, persons with STI and sex workers not using condoms.

This correlates with the current epidemic pattern in Italy, where some 4000–10000 (according to the different models and data sources) new HIV infections are estimated to occur yearly: the highest estimated number of recent infections is among MSM [17,18], and the highest HIV prevalences have been observed in MSM, persons with a current STI, and sex workers [19].

Regardless of their attitude, respondents expressed the concern that widespread access to PrEP could lead to an increased incidence of HIV and STI among at-risk populations, unintended toxicity and the emergence of resistant strains, consistently with other studies [13,15,20]. However, only toxicity concerns were significantly more frequent among specialists showing a negative attitude towards PrEP. As for financial sustainability, although deemed too expensive, most specialists advocated NHS support of PrEP costs to ensure equity of access, consistently with other studies where healthcare providers recognized cost as a major barrier for patients [13], and wished public programmes to pay for PrEP if patients cannot afford it [15].

Currently, PrEP is considered cost-effective when targeted to high-risk populations with an annual incidence >2% [21], as evidenced for MSM in Italy [22]; further targeting based on risk factors (younger age, number of annual partners, not being tested for HIV annually) could help reducing PrEP financial impact.

The main predictor of a negative attitude towards PrEP prescription seems to be the scarce propensity to offer or inform patients on PEP, and more in general to consider ARV as a tool to prevent HIV transmission. Specialists with a negative attitude believe behavioural interventions to be more effective than PrEP, and feel uncomfortable when prescribing drugs for new indications unless there is clear evidence of benefit. The results of a survey conducted at the conclusion of the 2012 International Association of Physicians in AIDS Care (IAPAC) meeting “Controlling the HIV Epidemic with Antiretrovirals” are in line with ours: over half of the summit attendees (56%) chose funding as the largest barrier to implementation of PrEP; most participants (70%) said that in their country PrEP will be used only in selected cases, and mostly (91%) following formal PrEP guidelines [23].

Even in our study most HIV specialists would welcome guidelines on PrEP, and the start of a clinical trial where their patients could be enrolled. Those with a negative attitude did not consider advisable that formal PrEP guidelines were issued (OR 0.30; 95% CI 0.13-0.66; p = 0.003).

When our survey was conducted only two guidance documents were available, from the Centers for Disease Control and Prevention (CDC) [24] and the British HIV Association/British Association for Sexual Health and HIV [25], mainly oriented to limit inappropriate prescription, respectively, among MSM and outside the context of a clinical research study.

Following FDA approval [26], and the availability of conclusive data from studies of oral PrEP among heterosexuals [6,7], the WHO released recommendations [27], and the CDC issued a second interim guidelines targeted to high-risk heterosexually active adults [28], both aimed at guiding clinicians on providing PrEP outside of the context of controlled clinical trials. So far, the European Medicines Agency (EMA) has issued a Reflection paper whose purpose is to highlight regulatory aspects regarding the preclinical and clinical development of PrEP [29].

Whether and how these documents might impact on HIV specialists’ attitude and concerns and on the European and Italian settings, will need monitoring and further reassessment.

Some of the barriers to PrEP prescription evidenced in our study will probably be addressed by future trials and open label studies [30].

Other concerns, such as adherence, risk disinhibition and the emergence of resistant strains, need further evidence on wider populations and longer periods of observation.

It is encouraging that HIV specialists, already loaded with their current activities, strongly disagree with a lack of time to engage in prevention counselling and clinical monitoring of individuals on PrEP, differently from what observed in other studies [20]. This is somehow consistent with the high frequency with which PEP information and treatment is provided in this group, and in accordance with similar observations both in HIV specialists and in generalists [15].

Training and providing continuing education to the health workforce is another significant challenge. In our survey, one third of interviewed HIV specialists reported a poor knowledge of PrEP; also during the focus group, participants reported unsatisfactory knowledge of PrEP trial results. Even if we did not find a significant association with the reported attitude towards PrEP prescription, better knowledge has been associated with a higher willingness to prescribe PrEP [13,15].

Our results may not apply to the whole population of Italian HIV specialists.

First of all the participation rate was weak (40%), and no data have been collected allowing to compare anonymous respondents with non-respondents in order to characterize the possible participation bias. Participants represent a convenience sample, obtained using two different recruitment methods (i.e. participation in HIV educational courses versus participation in the online survey). The online sample is composed only of members of the Italian Society of Infectious Diseases, which collects most, but not all, of the Italian infectious disease/HIV specialists.

Social desirability bias may have influenced the answers to the questionnaires provided at the educational HIV courses, although anonymous. This could explain at least in part the significantly more positive attitude towards PrEP prescription among respondent specialists who participated in the HIV courses.

Conversely, previous studies have demonstrated that online data collection has the potential to limit this bias and result in more honest and accurate responses [31,32].

Therefore, we performed a sub-analysis only of the online sample: the results we obtained were consistent with those of the overall population.

Moreover, the questionnaire did not collect any personal information about the physician (e.g. marital status, being HIV positive or having close friends at risk of or with HIV infection, religious faith or political beliefs, etc.) which could influence specialists’ attitude toward PrEP in higher proportions than the professional characteristics tested in the model [33,34]; indeed, the multivariate model only explains 17.2% of the variance. Therefore, further studies are needed to identify other predictors of PrEP prescription apart from PEP provision.

## Conclusion

In conclusion, the majority of respondent HIV specialists would prescribe PrEP in specific situations. However, conflicting attitudes appear evident, in line with the results of a New England Journal of Medicine polling on PrEP, and reflect uncertainties within the medical and public health communities over which approaches will most effectively control the global spread of HIV [35]. Whether additional scientific evidence and enhanced knowledge could modify attitudes needs monitoring and further investigation.

Identifying the populations at high risk for HIV infection and for whom PrEP use would be most effective, and linking and retaining them in care, is one of the emerging challenges. HIV specialists seem to be willing, pending further evidence from ongoing studies and guidance from normative bodies, to promote PrEP within multiple prevention strategies among vulnerable populations.

## Competing interests

The authors declare that they have no competing interest.

## Authors’ contributions

All authors were involved in the design of the study, and contributed to drafts of this manuscript. VP conceived the study and with GI was responsible for overall coordination. AP and VP conducted the Focus Group, and developed the questionnaire. GDC and VP interpreted the data, and took the lead on writing the report. AP and PP managed the data and conducted the statistical analysis. All authors read and approved the final manuscript.

## Pre-publication history

The pre-publication history for this paper can be accessed here:

http://www.biomedcentral.com/1471-2334/13/217/prepub

## Supplementary Material

Additional file 1 Questionnaire: PrEPscription among HIV specialists - Apr2012. Click here for file
