# Supplementary material for: Attitude towards antiretroviral pre-exposure prophylaxis (PrEP) prescription among HIV specialists
Source: BMC Infect Dis. 2013 May 14;13:217. doi: 10.1186/1471-2334-13-217 (PMC3658955; doi:10.1186/1471-2334-13-217)
Supplement: Additional file 1 — Questionnaire: PrEPscription among HIV specialists - Apr2012. [file 1471-2334-13-217-S1.pdf]

Pre-Exposure Prophylaxis (PrEP) Prescription among HIV Specialists

1. Workplace

☐1 Non teaching hospital ☐2 University/Research institute ☐3 Other *(please specify)*______

2. Region ……………………………….

Demographic characteristics and work history

3. Age Class

☐1 <30
☐2 31-40
☐3 41-50
☐4 51-60
☐5 >60

4. Gender

☐1 Male
☐2 Female

5. Length of service *(years)*

☐1 <5
☐2 5-9
☐3 10-14
☐4 15-19
☐5 >20

6. Prevailing activity
(one choice)

☐1 Ward/Inpatients
☐2 Outpatient clinic/Day hospital
☐3 Other *(please specify)*__________

7. HIV-infected persons currently followed

☐0 0
☐1 1-5
☐2 6-20
☐3 21-50
☐4 51-100
☐5 > 100

8. HIV tests prescribed in the last month

☐0 0
☐1 1-5
☐2 6-20
☐3 21-50
☐4 51-100
☐5 > 100

9. When did you start treating patients with HIV infection?

|__|__|__|__|
Year

10. Did you prescribe antiretrovirals to HIV-uninfected persons to prevent HIV infection?
(check all that apply)

☐1 Yes, after occupational exposure (Post-exposure prophylaxis - PEP)
☐2 Yes, after non occupational exposure (sexual or injection drug use - nPEP)
☐3 Yes, before any exposure (Pre-Exposure Prophylaxis - PrEP)
☐4 No, never

11. In your clinical practice, do you inform individuals in advance of the possibility of using antiretroviral post-exposure prophylaxis in case of sexual exposure?
(one choice)

☐1 No

☐2 Yes

12. How would you rate your knowledge about PrEP?

☐1 Poor
☐2 Sufficient
☐3 Good

13. Based on currently available evidence on PREP, do you believe…
(check all that apply)

	Advisable	Inadvisable
a. Participation in a multicenter trial	O1	O2
a. International/national PrEP guidelines	O1	O2
b. Internal protocol	O1	O2

14. Based on currently available evidence, do you think PrEP should be offered, and in case to whom?

☐1 No
☐2 Yes

In case of: *(check all that apply)*

Injection drug user	☐1	
	NOT using condoms	***Always***
Men who have sex with men (MSM)	☐2	☐3
Persons with sexually transmitted infections	☐4	☐5
Persons with multiple partners	☐6	☐7
Sex workers/Transactional sex	☐8	☐9

Offer PrEP to HIV-uninfected partner in serodiscordant couples: *(check all that apply)*

	Viremic partner	Always
Men in heterosexual couples…	☐10	☐11
Women in heterosexual couples…	☐12	☐13
Men in MSM couples…	☐14	☐15
Women for conception…	☐16	☐17
Men for conception…	☐18	☐19

15. Do you think the costs of PrEP should be sustained by the NHS?
(one choice)

☐1 Yes, always
☐2 No, entirely sustained by the patient
☐3 Yes, but partially: shared with the patient on an income basis
☐4 Yes, but only in case of…*(please specify)*___________________________

16. In the case of HIV positive patients in serodiscordant couples who do not meet the criteria for starting treatment, which of the following options would you choose to protect the negative partner?
(one choice)

☐1 ARV to the positive patient
☐2 PrEP to the uninfected partner
☐3 Treat both partners
☐4 Only counselling for safe sex

17. State whether you agree or disagree with the following statements….

	Agree	Disagree
a. I am concerned that PrEP will not be 100% effective	O_1	O_2
b. I am concerned about the potential side effects of PrEP	O_1	O_2
c. I feel uncomfortable prescribing drugs for new indications unless there are clear evidences	O_1	O_2
d. I am concerned about a low adherence to PrEP	O_1	O_2
e. I do not have time to engage in prevention counselling and PrEP monitoring	O_1	O_2
f. The use of PrEP will cause patients to engage in riskier behaviours	O_1	O_2
g. The provision of PrEP will result in an increase in sexually transmitted disease incidence among patients	O_1	O_2
h. Encourage access to testing and care for HIV infection are more effective measures	O_1	O_2
i. Non-biomedical HIV prevention interventions (behavioural) are more effective than PrEP	O_1	O_2
j. Non-biomedical HIV prevention interventions (behavioural) are safer than PrEP	O_1	O_2
k. The use of PrEP will result in less frequent HIV testing among patients	O_1	O_2
l. PrEP is too costly	O_1	O_2
m. The use of antiretrovirals for prevention will select for, and disseminate, antiretroviral drug resistance	O_1	O_2

Thank you for your collaboration
